# 2-(2-Iodo­benzenesulfonamido)acetic acid

**DOI:** 10.1107/S1600536809005248

**Published:** 2009-02-18

**Authors:** Muhammad Nadeem Arshad, Islam Ullah Khan, Muhammad Shafiq, Azam Mukhtar

**Affiliations:** a Materials Chemistry Laboratory, Department of Chemistry, GC University, Lahore, Pakistan; b Institute for Chemical Technology and Analytics, Vienna University of Technology, Vienna, Austria

## Abstract

The title compound, C_8_H_8_INO_4_S, is a halogenated sulfon­amide, a medicinally important class of organic compounds. In the crystal structure, inter­molecular O—H⋯O hydrogen bonds involving the carboxylic acid groups form characteristic centrosymmetric dimers. These dimers are further linked through centrosymmetric dimeric N—H⋯O inter­actions involving the amido H atom and a sulfonyl O atom. This leads to the formation of a ribbon-like polymer structure propagating in the *b* direction.

## Related literature

For background on sulfonamides, or sulfa drugs, see: Pandya *et al.* (2003 ▶). For the structure of the non-halogenated analogue, see: Arshad *et al.* (2008*b* ▶). For the synthesis of the title compound, see: Deng & Mani (2006 ▶). For details of related structures: see Arshad *et al.* (2008*a* ▶,*c* ▶). For background on related thia­zine heterocycles, see: Arshad *et al.* (2008*d* ▶). For standard bond-length data, see: Allen *et al.* (1987 ▶).
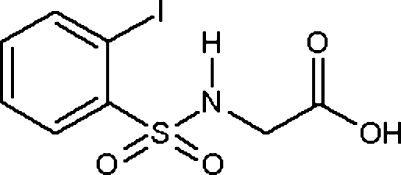


## Experimental

### 

#### Crystal data

C_8_H_8_INO_4_S
*M* *_r_* = 341.11
Triclinic, 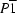

*a* = 5.5877 (2) Å
*b* = 8.0145 (2) Å
*c* = 12.3584 (4) Å
α = 80.923 (2)°
β = 83.398 (2)°
γ = 88.038 (2)°
*V* = 542.81 (3) Å^3^
*Z* = 2
Mo *K*α radiation
μ = 3.14 mm^−1^
*T* = 296 K
0.22 × 0.10 × 0.06 mm

#### Data collection

Bruker Kappa APEXII CCD diffractometer
Absorption correction: multi-scan (*SADABS*; Bruker, 2005 ▶) *T*_min_ = 0.691, *T*_max_ = 0.834
11492 measured reflections
2691 independent reflections
2297 reflections with *I* > 2σ(*I*)
*R*_int_ = 0.028

#### Refinement

*R*[*F* ^2^ > 2σ(*F* ^2^)] = 0.030
*wR*(*F* ^2^) = 0.086
*S* = 1.02
2691 reflections
137 parameters
H-atom parameters constrained
Δρ_max_ = 1.43 e Å^−3^
Δρ_min_ = −1.09 e Å^−3^

### 

Data collection: *APEX2* (Bruker, 2007 ▶); cell refinement: *SAINT* (Bruker, 2007 ▶); data reduction: *SAINT*; program(s) used to solve structure: *SHELXS97* (Sheldrick, 2008 ▶); program(s) used to refine structure: *SHELXL97* (Sheldrick, 2008 ▶); molecular graphics: *ORTEP-3 for Windows* (Farrugia, 1997 ▶) and *PLATON* (Spek, 2009 ▶); software used to prepare material for publication: *WinGX* (Farrugia, 1999 ▶) and *PLATON*.

## Supplementary Material

Crystal structure: contains datablocks I, global. DOI: 10.1107/S1600536809005248/su2093sup1.cif

Structure factors: contains datablocks I. DOI: 10.1107/S1600536809005248/su2093Isup2.hkl

Additional supplementary materials:  crystallographic information; 3D view; checkCIF report

## Figures and Tables

**Table 1:** Hydrogen-bond geometry (Å, °)

*D*—H⋯*A*	*D*—H	H⋯*A*	*D*⋯*A*	*D*—H⋯*A*
N1—H1N⋯O1^i^	0.86	2.47	3.142 (3)	135
O2—H2O⋯O1^ii^	0.82	1.86	2.676 (4)	176

Symmetry codes: (i) 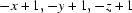; (ii) 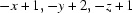.

**Fig. 1.**
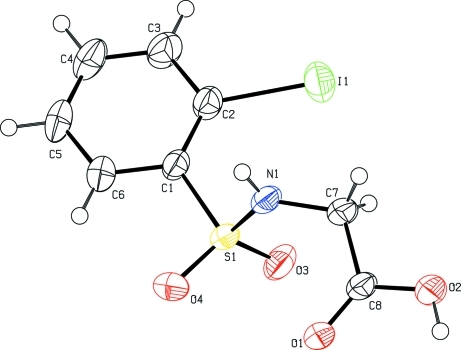
Molecular structure of the title compound showing the atom labelling scheme. The thermal ellipsoids are drawn at the 50% probability level.

**Fig. 2.**
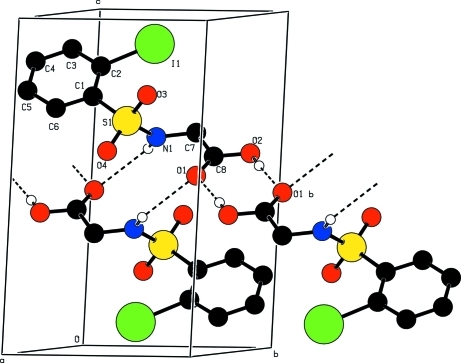
Crystal packing of the title compound, viewed along the axis, showing the intermolecular hydrogen bonds as dashed lines.

## supplementary crystallographic information

### Comment

Sulfonamides, commonly known as sulfa drugs, belong to a class of organic compounds which have widespread applications in Medicinal Chemistry for curing bacterial infections (Pandya *et al.*, 2003). As a continuation of our studies on the synthesis of thiazine related compounds (Arshad *et al.*, 2008*a*,*b*,*c*,*d*) we present here the structure of the title compound.

The molecular structure of the title compound is illustrated in Fig. 1. We have previously reported on the crystal structures of the non-halogenated analogue of the title compound, 2-(phenylsulfonamido)acetic acid, (Arshad *et al.*, 2008*b*), and the K^+^ and Na^+^ salts of 2-iodobenzenesulfonates (Arshad *et al.*, 2008*a*,*c*). The bond lengths and angles in the title compound are similar to those reported there and are within normal ranges (Allen *et al.*, 1987).

In the crystal structure intermolecular O—H···O hydrogen bonds involving the carboxylic acid groups form characteristic centrosymmetric dimers. These dimers are further linked through centrosymmetric intermolecular N—H···O interactions involving the amido H atoms and a sulfonyl O atom (Fig. 2). This leads to the formation of a ribbon-like polymer structure propagating along the *b* axis. This arrangement is very similar to that observed in the non-halogenated analogue mentioned above.

### Experimental

The title compound was synthesized following the literature method (Deng & Mani, 2006). Glycine methyl ester hydrochloride (207 mg, 1.653 mmol) was dissolved in distilled water (5 ml). The pH of the solution was maintained at 8–9 using 1*M*, Na_2_CO_3_ solution. 2-Iodo benzene sulfonyl chloride (500 mg, 1.653 mmol) was then added to the solution, which was stirred at room temperature until all the 2-iodo benzene sulfonyl chloride had been consumed. During the reaction the pH was again strictly maintained at 8–9 using 1*M*, Na_2_CO_3_. On completion of the reaction the pH was adjusted 1–2, using 1N HCl under vigorous stirring. The precipitate obtained was filtered off, washed with distilled water and dried. Crystals of the title compound were obtained by recrystallisation from methanol.

### Refinement

The O, N and C-bound H atoms were included in calculated positions and treated as riding: O—H = 0.82 Å, N—H = 0.86 Å, C—H = 0.93–0.97 Å, with *U*_iso_(H) = 1.5*U*_eq_(parent O atom) and = 1.2*U*_eq_(parent C and N atom).

### Crystal data

**Table:**

C_8_H_8_INO_4_S	*Z* = 2
*M**_r_* = 341.11	*F*(000) = 328
Triclinic, *P*1	*D*_x_ = 2.087 Mg m^−^^3^
Hall symbol: -P 1	Mo *K*α radiation, λ = 0.71073 Å
*a* = 5.5877 (2) Å	Cell parameters from 6313 reflections
*b* = 8.0145 (2) Å	θ = 2.6–28.3°
*c* = 12.3584 (4) Å	µ = 3.14 mm^−^^1^
α = 80.923 (2)°	*T* = 296 K
β = 83.398 (2)°	Needle-like, light brown
γ = 88.038 (2)°	0.22 × 0.10 × 0.06 mm
*V* = 542.81 (3) Å^3^	

### Data collection

**Table:**

Bruker Kappa APEXII CCD diffractometer	2691 independent reflections
Radiation source: fine-focus sealed tube	2297 reflections with *I* > 2σ(*I*)
graphite	*R*_int_ = 0.028
φ and ω scans	θ_max_ = 28.3°, θ_min_ = 2.6°
Absorption correction: multi-scan (*SADABS*; Bruker, 2005)	*h* = −7→7
*T*_min_ = 0.691, *T*_max_ = 0.834	*k* = −10→10
11492 measured reflections	*l* = −16→16

### Refinement

**Table:**

Refinement on *F*^2^	Primary atom site location: structure-invariant direct methods
Least-squares matrix: full	Secondary atom site location: difference Fourier map
*R*[*F*^2^ > 2σ(*F*^2^)] = 0.030	Hydrogen site location: inferred from neighbouring sites
*wR*(*F*^2^) = 0.086	H-atom parameters constrained
*S* = 1.02	*w* = 1/[σ^2^(*F*_o_^2^) + (0.0454*P*)^2^ + 0.6546*P*] where *P* = (*F*_o_^2^ + 2*F*_c_^2^)/3
2691 reflections	(Δ/σ)_max_ = 0.001
137 parameters	Δρ_max_ = 1.43 e Å^−^^3^
0 restraints	Δρ_min_ = −1.09 e Å^−^^3^

### Special details

**Table:**

Geometry. Bond distances, angles etc. have been calculated using the rounded fractional coordinates. All su's are estimated from the variances of the (full) variance-covariance matrix. The cell esds are taken into account in the estimation of distances, angles and torsion angles
Refinement. Refinement of *F*^2^ against ALL reflections. The weighted *R*-factor *wR* and goodness of fit *S* are based on *F*^2^, conventional *R*-factors *R* are based on *F*, with *F* set to zero for negative *F*^2^. The threshold expression of *F*^2^ > σ(*F*^2^) is used only for calculating *R*-factors(gt) *etc*. and is not relevant to the choice of reflections for refinement. *R*-factors based on *F*^2^ are statistically about twice as large as those based on *F*, and *R*- factors based on ALL data will be even larger.

### Fractional atomic coordinates and isotropic or equivalent isotropic displacement parameters (Å^2^)

**Table:**

	*x*	*y*	*z*	*U*_iso_*/*U*_eq_	
I1	0.10590 (5)	0.34414 (3)	0.90179 (2)	0.0537 (1)	
S1	0.59293 (13)	0.42594 (9)	0.68657 (7)	0.0327 (2)	
O1	0.5522 (4)	0.7909 (3)	0.5250 (2)	0.0405 (7)	
O2	0.2240 (4)	0.9320 (3)	0.5837 (3)	0.0506 (8)	
O3	0.6165 (5)	0.5362 (3)	0.7649 (2)	0.0447 (8)	
O4	0.7874 (4)	0.4121 (3)	0.6022 (2)	0.0448 (8)	
N1	0.3478 (5)	0.4818 (3)	0.6322 (2)	0.0348 (8)	
C1	0.5418 (5)	0.2165 (4)	0.7568 (3)	0.0319 (8)	
C2	0.3582 (6)	0.1730 (4)	0.8409 (3)	0.0376 (9)	
C3	0.3352 (8)	0.0057 (4)	0.8907 (3)	0.0496 (11)	
C4	0.4944 (8)	−0.1169 (4)	0.8574 (3)	0.0520 (13)	
C5	0.6787 (8)	−0.0735 (4)	0.7758 (4)	0.0514 (11)	
C6	0.7032 (7)	0.0926 (4)	0.7254 (3)	0.0434 (10)	
C7	0.2290 (6)	0.6417 (4)	0.6433 (3)	0.0371 (9)	
C8	0.3550 (6)	0.7946 (4)	0.5770 (3)	0.0346 (9)	
H1N	0.28640	0.41450	0.59550	0.0420*	
H2O	0.29800	1.01380	0.54930	0.0760*	
H3	0.21160	−0.02410	0.94690	0.0600*	
H4	0.47630	−0.22890	0.89050	0.0630*	
H5	0.78740	−0.15580	0.75430	0.0620*	
H6	0.82870	0.12170	0.67000	0.0520*	
H7A	0.21220	0.65480	0.72050	0.0440*	
H7B	0.06810	0.63890	0.62140	0.0440*	

### Atomic displacement parameters (Å^2^)

**Table:**

	*U*^11^	*U*^22^	*U*^33^	*U*^12^	*U*^13^	*U*^23^
I1	0.0578 (2)	0.0421 (2)	0.0529 (2)	0.0132 (1)	0.0143 (1)	0.0003 (1)
S1	0.0304 (3)	0.0232 (3)	0.0442 (4)	−0.0049 (3)	−0.0027 (3)	−0.0042 (3)
O1	0.0375 (12)	0.0252 (10)	0.0551 (14)	−0.0047 (9)	0.0058 (10)	−0.0016 (9)
O2	0.0413 (13)	0.0247 (11)	0.0772 (18)	0.0014 (9)	0.0106 (12)	0.0057 (11)
O3	0.0498 (14)	0.0282 (11)	0.0602 (16)	−0.0014 (10)	−0.0147 (12)	−0.0130 (10)
O4	0.0337 (11)	0.0411 (13)	0.0565 (15)	−0.0076 (10)	0.0046 (10)	−0.0034 (11)
N1	0.0353 (13)	0.0217 (11)	0.0466 (15)	−0.0039 (9)	−0.0051 (11)	−0.0016 (10)
C1	0.0348 (15)	0.0227 (12)	0.0391 (16)	0.0011 (11)	−0.0065 (12)	−0.0059 (11)
C2	0.0442 (17)	0.0279 (14)	0.0394 (17)	0.0028 (12)	−0.0029 (13)	−0.0035 (12)
C3	0.063 (2)	0.0326 (17)	0.048 (2)	−0.0015 (15)	0.0038 (17)	0.0022 (14)
C4	0.075 (3)	0.0260 (16)	0.054 (2)	0.0038 (16)	−0.009 (2)	−0.0024 (14)
C5	0.062 (2)	0.0287 (16)	0.065 (2)	0.0135 (15)	−0.0084 (19)	−0.0137 (16)
C6	0.0454 (18)	0.0322 (16)	0.052 (2)	0.0049 (13)	0.0002 (15)	−0.0102 (14)
C7	0.0333 (15)	0.0247 (13)	0.0495 (18)	−0.0027 (11)	0.0011 (13)	0.0018 (12)
C8	0.0343 (15)	0.0229 (13)	0.0449 (17)	−0.0033 (11)	−0.0040 (13)	−0.0002 (12)

### Geometric parameters (Å, °)

**Table:**

I1—C2	2.094 (3)	C2—C3	1.388 (5)
S1—O3	1.429 (3)	C3—C4	1.382 (5)
S1—O4	1.431 (2)	C4—C5	1.369 (6)
S1—N1	1.613 (3)	C5—C6	1.382 (5)
S1—C1	1.780 (3)	C7—C8	1.509 (5)
O1—C8	1.212 (4)	C3—H3	0.9300
O2—C8	1.310 (4)	C4—H4	0.9300
O2—H2O	0.8200	C5—H5	0.9300
N1—C7	1.442 (4)	C6—H6	0.9300
N1—H1N	0.8600	C7—H7A	0.9700
C1—C6	1.389 (5)	C7—H7B	0.9700
C1—C2	1.385 (5)		
			
O3—S1—O4	118.92 (15)	C1—C6—C5	120.5 (4)
O3—S1—N1	106.70 (15)	N1—C7—C8	115.3 (3)
O3—S1—C1	109.54 (16)	O1—C8—O2	124.4 (3)
O4—S1—N1	110.20 (14)	O1—C8—C7	124.5 (3)
O4—S1—C1	105.65 (15)	O2—C8—C7	111.1 (3)
N1—S1—C1	105.02 (13)	C2—C3—H3	120.00
C8—O2—H2O	109.00	C4—C3—H3	120.00
S1—N1—C7	121.8 (2)	C3—C4—H4	120.00
C7—N1—H1N	119.00	C5—C4—H4	120.00
S1—N1—H1N	119.00	C4—C5—H5	120.00
C2—C1—C6	119.5 (3)	C6—C5—H5	120.00
S1—C1—C6	116.4 (3)	C1—C6—H6	120.00
S1—C1—C2	124.1 (2)	C5—C6—H6	120.00
I1—C2—C1	124.6 (2)	N1—C7—H7A	108.00
I1—C2—C3	116.0 (3)	N1—C7—H7B	108.00
C1—C2—C3	119.5 (3)	C8—C7—H7A	108.00
C2—C3—C4	120.5 (4)	C8—C7—H7B	108.00
C3—C4—C5	120.1 (3)	H7A—C7—H7B	107.00
C4—C5—C6	120.0 (4)		
			
O3—S1—N1—C7	15.3 (3)	C6—C1—C2—I1	178.1 (3)
O4—S1—N1—C7	−115.1 (3)	C6—C1—C2—C3	−1.5 (5)
C1—S1—N1—C7	131.6 (3)	S1—C1—C6—C5	179.6 (3)
O3—S1—C1—C2	54.6 (3)	C2—C1—C6—C5	1.3 (5)
O3—S1—C1—C6	−123.6 (3)	I1—C2—C3—C4	−179.3 (3)
O4—S1—C1—C2	−176.2 (3)	C1—C2—C3—C4	0.3 (6)
O4—S1—C1—C6	5.6 (3)	C2—C3—C4—C5	0.9 (6)
N1—S1—C1—C2	−59.7 (3)	C3—C4—C5—C6	−1.1 (6)
N1—S1—C1—C6	122.1 (3)	C4—C5—C6—C1	−0.1 (6)
S1—N1—C7—C8	72.2 (4)	N1—C7—C8—O1	−6.5 (5)
S1—C1—C2—I1	0.0 (4)	N1—C7—C8—O2	174.0 (3)
S1—C1—C2—C3	−179.6 (3)		

### Hydrogen-bond geometry (Å, °)

**Table:**

*D*—H···*A*	*D*—H	H···*A*	*D*···*A*	*D*—H···*A*
N1—H1N···O1^i^	0.86	2.47	3.142 (3)	135
O2—H2O···O1^ii^	0.82	1.86	2.676 (4)	176

Symmetry codes: (i) −*x*+1, −*y*+1, −*z*+1; (ii) −*x*+1, −*y*+2, −*z*+1.
